# Carcinoid Heart Disease: Diagnostic Value of Cardiac MRI in a Patient With Metastatic Small‑Intestinal Neuroendocrine Tumor

**DOI:** 10.5334/jbsr.4191

**Published:** 2026-02-13

**Authors:** Simon Kaessner, Thiebault Saveyn, Benjamin Leenknegt

**Affiliations:** 1 UZ Brussel, 1090 Jette, Belgium; 2 AZ Sint‑Lucas Gent, 9000 Gent, Belgium

**Keywords:** carcinoid heart disease, cardiac magnetic resonance imaging (CMR)

## Abstract

Carcinoid heart disease (CHD) is a serious and potentially life‑limiting complication of neuroendocrine tumors (NETs), resulting from prolonged systemic exposure to serotonin and other vasoactive substances, leading to fibrotic valvular degeneration. We report the case of a 71‑year‑old woman with metastatic small‑intestinal NET and associated carcinoid syndrome presenting with progressive exertional intolerance. Transthoracic echocardiography revealed severe tricuspid and moderate pulmonary regurgitation with right‑sided ventricular dilation. Cardiac MRI confirmed regurgitation and right‑heart remodeling consistent with CHD.

*Teaching point:* This case highlights the diagnostic value of multimodal imaging, particularly cardiac MRI, in confirming and characterizing CHD in patients with metastatic NETs.

## Introduction

Carcinoid tumors are rare, slow‑growing neuroendocrine tumors (NETs) arising from hormone‑producing enteroendocrine cells, most commonly enterochromaffin cells of the gastrointestinal tract or their counterparts in other organs. These tumors secrete bioactive amines and peptides, such as serotonin and tachykinins, which are largely inactivated by the liver. When the secretion exceeds the hepatic capacity for degradation, they enter the systemic circulation, and systemic manifestations known as carcinoid syndrome may develop [1, 2]. Approximately 20%–40% of patients with carcinoid syndrome develop carcinoid heart disease (CHD) [2, 3].

This case illustrates how multimodal imaging plays an essential role in enabling timely diagnosis and optimal management of CHD in patients with metastatic neuroendocrine tumors.

## Case Report

**Figure 1:**
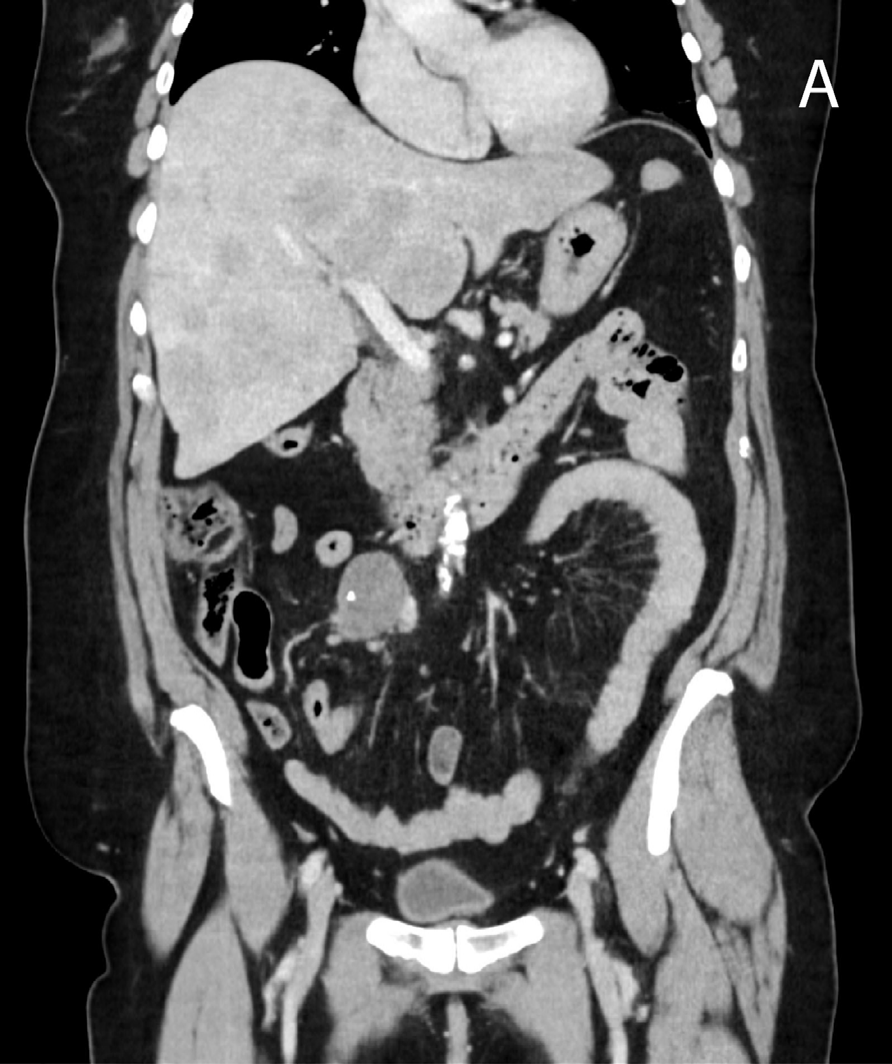

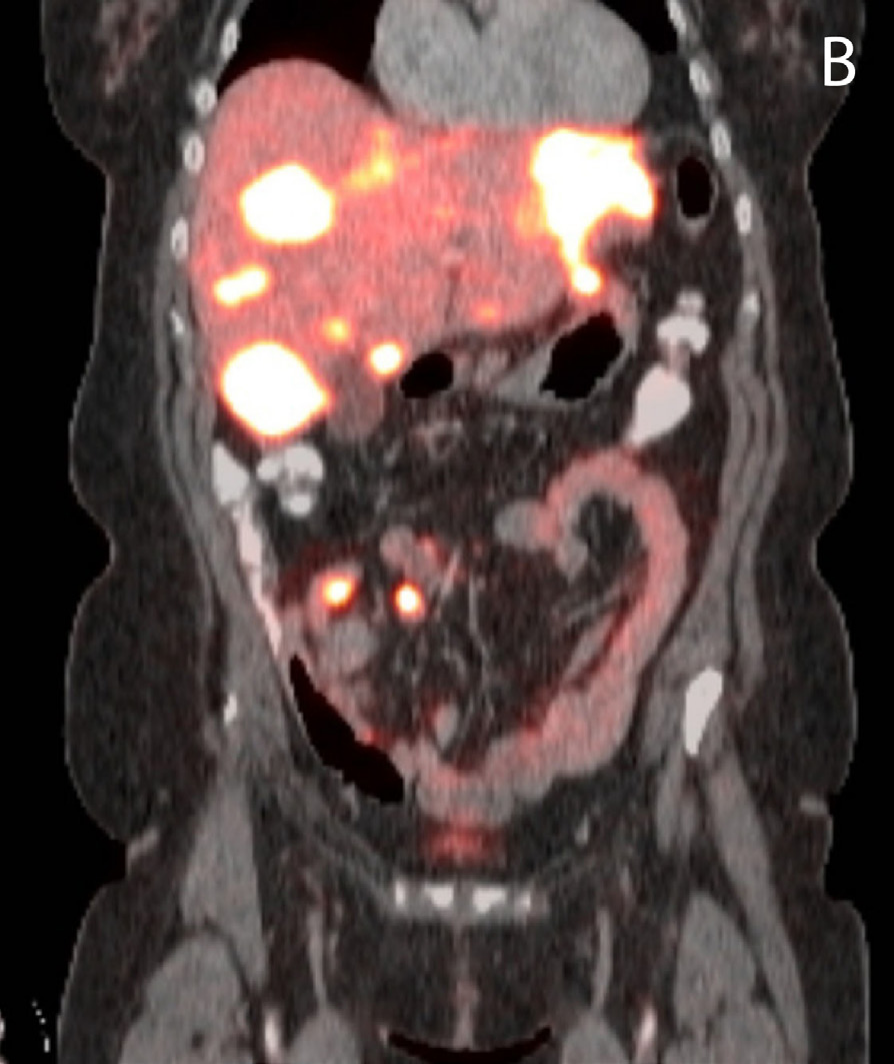
Primary small intestine NET with diffuse liver metastases: **(A)** coronal contrast‑enhanced CT shows a hypodense mesenterial mass (arrow) with central calcifications. **(B)** Al18F‑NOTA‑Octreotide PET‑CT shows multiple hyperenhancing liver nodules.

**Figure 2:**
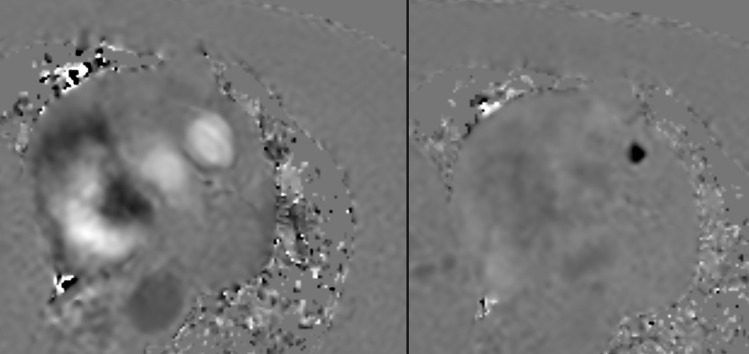
Pulmonary valve regurgitation of 30%: axial phase‑contrast (Q‑flow) cardiac MRI showing retrograde flow into the right ventricle during diastole.

**Figure 3:**
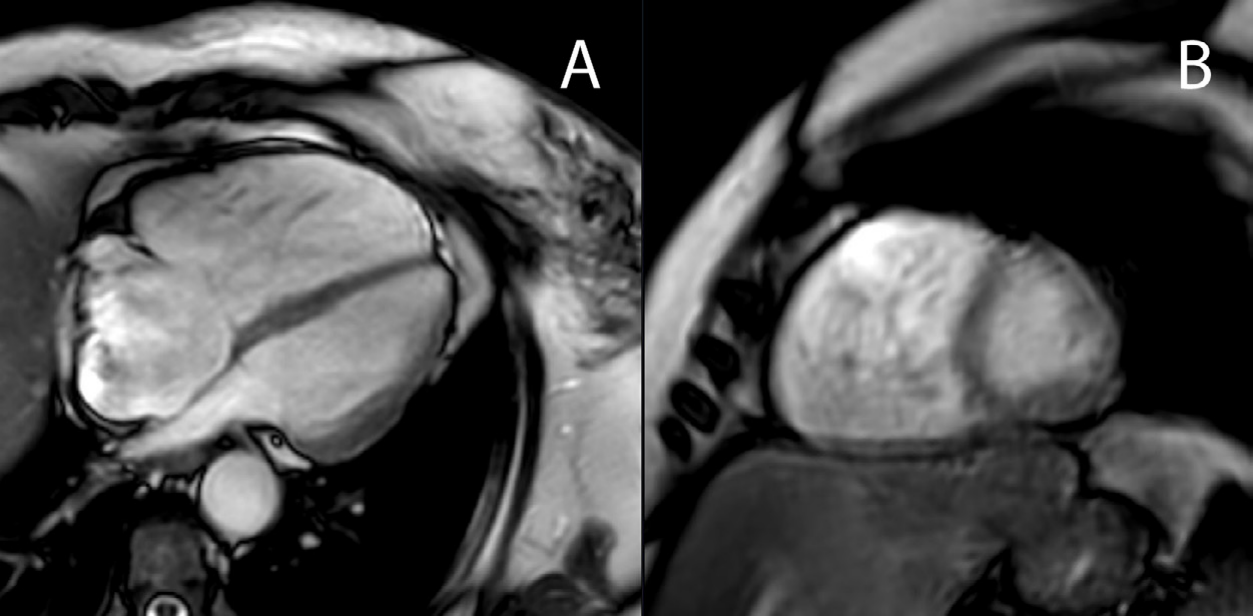
Right heart dilation and diastolic ‘D‑shaping’: axial **(A)** and sagittal **(B)** cine cardiac MR images showing dilation of the right atrium and ventricle with flattening of the interventricular septum ‘D‑shaping’ during diastole.

A 71‑year‑old woman, with a history of liver‑ and lymph node‑metastasized small‑intestinal NET (Figure 1) and known carcinoid syndrome, was referred for progressive exertional intolerance and fatigue. Transthoracic echocardiography revealed a right‑sided valvular dysfunction with a markedly dilated right atrium and slightly reduced systolic function. On cardiac MRI, pulmonary valve regurgitation of 11 mL or 30% (Figure 2) was demonstrated. The right atrium and ventricle were dilated, and there was a diastolic D‑shaping of the interventricular septum (Figure 3). The values on T1 mapping of the left ventricular myocardium were slightly elevated. Given the patient’s known carcinoid syndrome, findings on cardiac MRI were consistent with CHD.

## Discussion

CHD is a significant and potentially life‑limiting complication of NETs. If present, CHD has a more profound impact on the patient’s prognosis than the local tumor growth. Prolonged exposure to tumor‑secreted substances, particularly serotonin, induces valvular fibrosis through activation of 5‑HT_2_B receptors and transforming growth factor‑β pathways, resulting in thickened, retracted valve cusps and plaque deposition [1, 2]. This process predominantly affects the right‑sided cardiac valves, although left‑sided involvement can occur in the presence of a patent foramen ovale or exceptionally high serotonin levels [3]. Clinically, patients often present with nonspecific fatigue or dyspnea; therefore, the diagnosis requires a high index of suspicion in those with carcinoid syndrome [2]. Echocardiography is able to identify thickened, immobile valve leaflets and assess regurgitation severity [1]. However, as ultrasonography is operator‑ and patient‑dependent, pulmonary valve disease is frequently underestimated, and reduced right ventricular stroke volume leads to reduced regurgitant jet velocities [3]. Cardiac magnetic resonance imaging (CMR) provides a comprehensive qualitative and quantitative cardiac analysis, allowing precise measurement of regurgitant volumes and fractions, as well as assessment of right‑ventricular size, function, and remodeling [3]. In addition, tissue characterization techniques such as T1 mapping and late gadolinium enhancement can detect myocardial fibrosis or metastatic deposits [3]. These advantages make CMR increasingly valuable for comprehensive evaluation and for therapeutic planning [1, 3]. Treatment of patients with CHD focuses on controlling carcinoid activity with somatostatin analogues, relieving heart‑failure symptoms with diuretics, and potentially valve replacement when regurgitation is severe [2].

## Conclusion

CHD is a significant yet often underdiagnosed complication and prognostic determinant of metastatic NETs. This case emphasizes the need for high clinical suspicion in NET patients presenting with new or progressive cardiac symptoms. Cardiac MRI provides superior characterization of valvular and myocardial involvement, complementing echocardiography in diagnostic assessment. By illustrating the distinctive MRI features of CHD, this case report reinforces the role of multimodal imaging in early recognition and management of this rare entity.
